# Comment on: The environmental impact of intravenous antimicrobial therapies: a comparison of OPAT and inpatient administration care pathways

**DOI:** 10.1093/jacamr/dlaf192

**Published:** 2025-11-05

**Authors:** Philip Elders, Eva Cohen, Joppe Hovius, Kim Sigaloff

**Affiliations:** Department of Internal Medicine, Division of Infectious Diseases, Amsterdam UMC, Amsterdam, The Netherlands; Amsterdam Institute for Immunology & Infectious Diseases, Amsterdam UMC, Amsterdam, The Netherlands; Center for Infection and Molecular Medicine, Amsterdam UMC, Amsterdam, The Netherlands; Center for Sustainable Healthcare, Amsterdam UMC, Amsterdam, The Netherlands; Center for Sustainable Healthcare, Amsterdam UMC, Amsterdam, The Netherlands; Amsterdam Public Health, Quality of Care, Global Health, Amsterdam, The Netherlands; Amsterdam Reproduction and Development Research Institute, Amsterdam UMC, Amsterdam, The Netherlands; Department of Internal Medicine, Division of Infectious Diseases, Amsterdam UMC, Amsterdam, The Netherlands; Amsterdam Institute for Immunology & Infectious Diseases, Amsterdam UMC, Amsterdam, The Netherlands; Center for Infection and Molecular Medicine, Amsterdam UMC, Amsterdam, The Netherlands; Department of Internal Medicine, Division of Infectious Diseases, Amsterdam UMC, Amsterdam, The Netherlands; Amsterdam Institute for Immunology & Infectious Diseases, Amsterdam UMC, Amsterdam, The Netherlands

With great interest, we read the article by Cole *et al.* on the environmental impact of intravenous antimicrobial therapies.^1^ We commend the authors for addressing a highly urgent and underexplored area of research on the environmental sustainability of infectious disease care. The article contributes to the growing body of research estimating the environmental impact of care pathways,^2^ which can support both providers and patients to make climate-informed healthcare decisions.^3^

The authors of the article describe that outpatient services using elastomeric pump devices for intravenous antimicrobial therapy have a substantially lower environmental impact compared with conventional in-hospital intravenous administration. The study highlights this potential lower environmental impact as an additional advantage of outpatient antimicrobial therapy (OPAT), which is already recognized as providing safe and cost-effective care, while increasing patient autonomy and comfort, particularly when OPAT is self-administered.^4,5^ While we support this type of research, we would like to highlight four major issues regarding the methodology of this environmental impact assessment that warrant a cautious interpretation of the results.

First, a fundamental issue concerns the chosen functional unit of the study—10 adult patients for whom OPAT is considered a viable treatment option—which appears to be different in the in-hospital care pathway compared with the OPAT care pathways. The data used for the in-hospital pathway do not reflect the chosen functional unit of patients for whom OPAT is considered a viable treatment option, since the authors used secondary data from the NHS Sustainable Healthcare Pathways Guidance^6^ to describe the in-hospital patients. These data are based on aggregated data for an average patient at a low intensity ward in the UK for 24 hours. This ‘average’ patient is by definition different from an OPAT-eligible patient, as they are acutely ill and have to stay on an inpatient ward requiring more extensive care. Therefore, they are not comparable in terms of consumable use and nursing requirements to a patient who is stable enough to be discharged with OPAT. As a result, even though the article claims to compare the same patients (functional unit), the underlying data for the in-hospital scenario represent a fundamentally different patient population. This leads to an inappropriate comparison and likely overestimates the environmental impact of the in-hospital scenario.

Second, the system boundaries for the in-hospital scenario are not comparable to the OPAT scenarios. While food consumption, building energy use and staff travel are included in the in-hospital system boundaries by using secondary data,^6^ food and building energy use is excluded in the OPAT scenarios and staff travel is omitted from the outpatient OPAT scenario. Although it is insightful to consider the environmental footprint of the care facilities and associated energy use, consumable use and travel movements of staff and patients in environmental impact assessments of care pathways,^7^ inconsistent system boundaries lead to unfair comparisons: in this case amplifying the environmental impact of the in-hospital scenario.^8^

Third, the authors introduce a methodological inconsistency by double counting consumables in the in-hospital scenario, since the vascular access insertion, drug reconstitution, drug administration and blood tests are already included as (average) consumables in the NHS Sustainable Healthcare Pathways Guidance data.^6^ As a result, the environmental impact of the in-hospital scenario is again likely to be overestimated.

Fourth, the authors state they performed a materiality assessment to identify the most impactful components of the care pathways and prioritize detailed data collection accordingly.^1^ However, the authors, who are affiliated with a company producing elastomeric pump devices, chose to perform a detailed life cycle assessment only of the elastomeric pump that has a relatively low impact while relying on uncertain, high-level secondary data for inpatient hospital care that has the largest impact. This may have led to truncation errors,^8^ introducing omissions of the environmental impact caused by simplifying or cutting off parts of the analysis.

In conclusion, the study by Cole *et al.*^1^ makes an important contribution by reporting on the environmental impact of infectious disease care. Yet, the article likely largely overestimates the environmental impact of the in-hospital scenario compared with the OPAT scenarios due to methodological inconsistencies. Although the authors do not claim to conduct a full life cycle assessment, the same methodological principles still apply.^9^ In future environmental impact assessment studies, it is important that the goal and scope are clearly specified, that primary or secondary data serve the same, precisely defined functional unit, that consistent system boundaries are chosen, that uncertainties are accounted for by sensitivity analyses and that the potential consequences of methodological choices are accurately reflected on. This can provide healthcare professionals with a robust comparison to base their decisions and guidelines on.
